# Hepatitis B immunization for indigenous adults, Australia

**DOI:** 10.2471/BLT.16.169524

**Published:** 2016-09-16

**Authors:** Andre Louis Wattiaux, J Kevin Yin, Frank Beard, Steve Wesselingh, Benjamin Cowie, James Ward, Kristine Macartney

**Affiliations:** a National Centre for Immunisation Research and Surveillance, Kerry Packer Building, Children’s Hospital at Westmead, corner Hawkesbury Rd and Hainsworth St, Westmead NSW 2145, Australia.; b South Australian Health and Medical Research Institute, Adelaide, Australia.; c WHO Collaborating Centre for Viral Hepatitis, Doherty Institute, Melbourne, Australia.

## Abstract

**Objective:**

To quantify the disparity in incidence of hepatitis B between indigenous and non-indigenous people in Australia, and to estimate the potential impact of a hepatitis B immunization programme targeting non-immune indigenous adults.

**Methods:**

Using national data on persons with newly acquired hepatitis B disease notified between 2005 and 2012, we estimated incident infection rates and rate ratios comparing indigenous and non-indigenous people, with adjustments for underreporting. The potential impact of a hepatitis B immunization programme targeting non-immune indigenous adults was projected using a Markov chain Monte Carlo simulation model.

**Findings:**

Of the 54 522 persons with hepatitis B disease notified between 1 January 2005 and 31 December 2012, 1953  infections were newly acquired. Acute hepatitis B infection notification rates were significantly higher for indigenous than non-indigenous Australians. The rates per 100 000 population for all ages were 3.6 (156/4 368 511) and 1.1 (1797/168 449 302) for indigenous and non-indigenous people respectively. The rate ratio of age-standardized notifications was 4.0 (95% confidence interval: 3.7–4.3). If 50% of non-immune indigenous adults (20% of all indigenous adults) were vaccinated over a 10-year programme a projected 527–549 new cases of acute hepatitis B would be prevented.

**Conclusion:**

There continues to be significant health inequity between indigenous and non-indigenous Australians in relation to vaccine-preventable hepatitis B disease. An immunization programme targeting indigenous Australian adults could have considerable impact in terms of cases of acute hepatitis B prevented, with a relatively low number needed to vaccinate to prevent each case.

## Résumé

**Objectif:**

Quantifier les disparités entre les populations autochtones et non autochtones d'Australie en ce qui concerne l'incidence de l'hépatite B et estimer l'impact potentiel d'un programme de vaccination contre l'hépatite B destiné aux adultes autochtones non immunisés.

**Méthodes:**

À l'aide des données nationales sur les nouveaux cas d'hépatite B signalés entre 2005 et 2012, nous avons estimé le taux d'incidence ainsi que les ratios des taux pour les personnes autochtones et les non autochtones et les avons comparés, en procédant à des ajustements pour tenir compte des sous-signalements. L'impact potentiel d'un programme de vaccination contre l'hépatite B destiné aux adultes autochtones non immunisés a été déterminé à l'aide d'un modèle de simulation de Monte Carlo par chaînes de Markov.

**Résultats:**

Sur les 54 522 cas d'hépatite B signalés entre le 1er janvier 2005 et le 31 décembre 2012, 1953 concernaient des personnes qui avaient récemment contracté la maladie. Le taux de signalement des hépatites B aiguës était nettement plus élevé pour les Australiens autochtones que pour les non autochtones. Le taux pour 100 000 habitants, tous âges confondus, était respectivement de 3,6 (156/4 368 511) et de 1,1 (1797/168 449 302) pour les autochtones et les non autochtones. Le ratio des taux de signalement standardisés selon l'âge était de 4,0 (intervalle de confiance de 95%: 3,7-4,3). Si 50% des adultes autochtones non immunisés (soit 20% de l'ensemble des adultes autochtones) étaient vaccinés, dans le cadre d'un programme sur 10 ans, 527 à 549 nouveaux cas d'hépatite B aiguë pourraient être évités.

**Conclusion:**

D'importantes inégalités sanitaires persistent entre les Australiens autochtones et non autochtones en ce qui concerne l'hépatite B évitable par la vaccination. Un programme de vaccination destiné aux adultes autochtones pourrait avoir un impact considérable sur la prévention des cas d'hépatite B aiguë, avec un nombre relativement faible de personnes à vacciner pour éviter l'apparition de nouveaux cas.

## Resumen

**Objetivo:**

Cuantificar la desigualdad en la incidencia de hepatitis B entre personas indígenas y no indígenas de Australia y estimar el posible impacto de un programa de inmunización contra la hepatitis B dirigido a adultos indígenas no inmunes.

**Métodos:**

Mediante el uso de información nacional sobre personas recién diagnosticadas con hepatitis B entre 2005 y 2012, se estimaron tasas de infección incidental y coeficientes de tasas comparando a personas indígenas y no indígenas, con ajustes para la ausencia informativa. Se estimó el posible impacto de un programa de inmunización contra la hepatitis B dirigido a adultos indígenas no inmunes utilizando un modelo de simulación de Montecarlo basado en las cadenas de Markov.

**Resultados:**

De las 54 522 personas con hepatitis B registradas entre el 1 de enero de 2005 y el 31 de diciembre de 2012, se adquirieron 1 953 nuevos contagios. Las tasas de registro de contagio de hepatitis B aguda fueron mucho mayores entre la población australiana indígena que entre la no indígena. Las tasas por cada 100 000 habitantes de todas las edades fueron de 3,6 (156/4 368 511) y de 1,1 (1 797/168 449 302) para los indígenas y los no indígenas respectivamente. El coeficiente de la tasa de los registros por edades fue de 4,0 (intervalo de confianza, IC, del 95%: 3,7–4,3). Si se vacunara el 50% de los adultos indígenas no inmunes (20% de todos los indígenas adultos) en un programa de 10 años, se evitarían de 527 a 549 nuevos casos de hepatitis B aguda.

**Conclusión:**

Sigue habiendo una gran desigualdad sanitaria entre los australianos indígenas y los no indígenas en relación con la hepatitis B evitable con vacunas. Un programa de inmunización dirigido a adultos indígenas australianos podría tener un impacto considerable para los casos de previsión de contagio de hepatitis B aguda, con un número relativamente bajo de vacunas necesarias para evitar cada caso.

## ملخص

**الغرض:**

قياس نسبة التفاوت في حدوث حالات التهاب الكبد من النمط **  **"B" بين السكان الأصليين وغير الأصليين في أستراليا، وتقدير التأثير المحتمل لبرنامج التحصين ضد التهاب الكبد من النمط **  **"B" الذي يستهدف السكان الأصليين البالغين الغير محصنين.

**الطريقة:**

قمنا بتقدير معدلات حدوث الإصابة ونسب المعدلات التي تقوم بالمقارنة بين السكان الأصليين والسكان غير الأصليين، مع إجراء التعديلات لمعالجة مشكلة النقص في الإبلاغ، باستخدام بيانات وطنية عن الأشخاص المصابين مؤخرًا بمرض التهاب الكبد من النمط "B" والذين تم الإخطار بأعدادهم في الفترة بين عامي 2005 و 2012. وتم توقّع التأثير المحتمل لبرنامج التحصين ضد مرض التهاب الكبد من النمط "B" الذي يستهدف السكان الأصليين البالغين الغير محصنين باستخدام نموذج المحاكاة لسلسلة ماركوف مونت كارلو.

**النتائج:**

كان عدد الإصابات الحديثة بالمرض يبلغ 1953 حالة من بين 54,522 شخصًا مصابًا بمرض التهاب الكبد من النمط "B" والتي تم الإخطار بأعدادها في الفترة ما بين 1 يناير/كانون الثاني 2005 و 31 ديسمبر/كانون الأول 2012. وكانت معدلات الإخطار بالإصابة بمرض التهاب الكبد من النمط "B" الحاد أعلى بكثير لدى السكان الأستراليين الأصليين عن السكان غير الأصليين. كانت المعدلات وفقًا لكل مجموعة تضم 100,000 من السكان من جميع الأعمار 3.6 (156/4,368,511‏) و 1.1 (1797/‏168,449,302) للسكان الأصليين وغير الأصليين على التوالي. وكانت نسبة معدلات الإخطار المقاسة بالعمر 4.0 (بنسبة أرجحية مقدارها 95‏%: 3.7–4.3). إذا تم تطعيم نسبة تبلغ 50‏% من السكان الأصليين البالغين غير المحصنين (20‏% من جميع السكان الأصليين البالغين) من خلال برنامج تطعيم يستمر لمدة 10 أعوام، فسيتم تجنب الإصابة بعدد حالات متوقع يتراوح من 527 إلى 549 حالة جديدة من حالات الإصابة بمرض التهاب الكبد من النمط "B".

**الاستنتاج:**

لا يزال هناك قدر كبير من عدم المساواة في مجال الرعاية الصحية بين الأستراليين الأصليين وغير الأصليين فيما يتعلق بمرض التهاب الكبد من النمط "B" الذي يمكن الوقاية منه بتوفير اللقاحات. ويمكن أن يكون لبرنامج التحصين الذي يستهدف الأستراليين الأصليين البالغين تأثير كبير من حيث حالات الإصابة بمرض التهاب الكبد من النمط "B" الحاد التي تمت الوقاية منها، من خلال وجود أعداد قليلة نسبيًا بحاجة للتطعيم للوقاية من الإصابة بالمرض.

## 摘要

**目的:**

量化澳大利亚土著和非土著居民之间的乙肝发病率差异，并估测以无免疫力土著成年人为目标群体的乙肝疫苗接种项目的潜在影响。

**方法:**

根据 2005 年至 2012 年间报告的乙肝病毒感染人群的国家数据，通过对比土著和非土著居民并针对漏报进行相应调整，我们估测了乙肝病毒感染率及其比值。 采用马尔科夫蒙特卡罗 (Markov chain Monte Carlo) 模拟模型呈现了以无免疫力土著居民为目标人群的乙肝疫苗接种项目的潜在影响。

**结果:**

2005 年 1 月 1 日 至 2012 年 12 月 31 日期间报告的 54 522 例乙肝病毒携带人群中，1953 例为新感染人群。 澳大利亚土著居民急性乙肝感染的呈报率要远远高出澳大利亚非土著居民。 每 100 000 万人口（不分年龄）当中，土著居民和非土著居民的乙肝感染率分别是 3.6 （156/4 368 511） 和 1.1 （1797/168 449 302）。 年龄标准化报告中的感染率比值为 4.0 （95% 置信区间： 3.7–4.3)。 如果 50% 的无免疫力土著成年人（占所有土著成年人的 20%）在 10 年期项目中接种乙肝疫苗，预计将会有 527-549 人避免感染急性乙肝。

**结论:**

在疫苗可预防乙型肝炎疾病方面，澳大利亚土著居民与非土著居民之间仍将存在巨大的健康不平等性。 在预防急性乙型肝炎方面，以澳大利亚土著居民为目标群体的疫苗接种项目可能会产生相当大的影响，只有相对较少数量的居民需要接种疫苗以预防急性乙型肝炎。

## Резюме

**Цель:**

Сопоставить неравномерность распространенности гепатита В среди коренных австралийцев и остального населения Австралии и оценить потенциальный вклад прививок от гепатита В в рамках программы по охвату не имеющих иммунитета коренных жителей.

**Методы:**

Используя национальные данные о лицах с выявленным гепатитом В за период с 2005 по 2012 год, мы оценили частоту случаев заражения и их соотношение среди коренного и остального населения с учетом неполноты сведений. Потенциальное влияние программы иммунизации против гепатита В, охватывающей не имеющих иммунитета взрослых коренных жителей Австралии, было спрогнозировано при помощи имитационной модели Монте-Карло по схеме марковской цепи.

**Результаты:**

Из 54 522 лиц с гепатитом В, выявленных в период между 1 января 2005 года и 31 декабря 2012 года, новые случаи инфицирования были отмечены у 1953 человек. Степень выявления острого гепатита В среди коренного населения Австралии была значительно выше, чем среди остальных жителей страны. В пересчете на 100 000 населения всех возрастов она составила 3,6 (156 случаев на 4 368 511 человек) для коренного и 1,1 (1797 случаев на 168 449 302 человека) для остального населения. Соотношение частот уведомлений со стандартизацией по возрасту составило 4,0 (95% доверительный интервал: 3,7–4,3). Если бы можно было вакцинировать 50% не имеющих иммунитета взрослых коренных жителей (20% всего взрослого коренного населения), то за 10 лет программы можно было бы предотвратить 527–549 случаев заболевания острым гепатитом В.

**Вывод:**

По-прежнему сохраняется значительное неравенство в области здравоохранения между коренным и остальным населением Австралии в заболеваемости предотвращаемым вакцинацией гепатитом В. Программа иммунизации, направленная на взрослое коренное население Австралии, могла бы оказать значительное влияние на предотвращение случаев острого гепатита В, при этом на предотвращение каждого случая требуется удельно вакцинировать относительно небольшое количество людей.

## Introduction

Australia in 2016 has a population of approximately 24 million people. Indigenous Australians (Aboriginal and Torres Strait Islander peoples) constitute 3.0% of the population and have a younger age structure than non-indigenous Australians, similar to that of low- and middle-income countries.^1^ Although health indicators for the general Australian population are comparable with those of other high-income countries, life expectancy and the burden of many diseases is greater among indigenous Australians.^2^^,^^3^ The health disparities between indigenous and non-indigenous Australians have been acknowledged by the Australian government, which has implemented a whole-of-government strategy, entitled *Closing the gap in indigenous disadvantage*, aiming to completely remove these disparities by the year 2030.^4^

Total viral hepatitis-related mortality in the Western Pacific Region of the World Health Organization (WHO) is now higher than deaths due to acquired immune deficiency syndrome, malaria and tuberculosis combined, constituting a critical public health challenge for the Region.^5^^,^^6^ In Australia, hepatitis B vaccination was recommended (but not nationally funded) for infants and adults in high-risk groups, including indigenous Australians, in the late 1980s.^7^ Universal vaccination of all infants commenced in the Northern Territory of Australia in 1990, followed by a funded national adolescent immunization programme starting in 1997. A funded universal infant hepatitis B immunization programme was introduced nationally in May 2000.^7^ The seroprevalence of hepatitis B virus surface antigen (HBsAg) in indigenous Australian adults was estimated to be 17% in a meta-analysis of studies conducted before 2000, thus meeting the WHO definition for high endemicity.^8^ Since then, HBsAg seroprevalence in indigenous Australians is estimated to have declined to 3.7% of the 548 366 population nationally in 2011.^9^ This is a WHO-defined intermediate level of endemicity, but is still more than 10 times the rate in non-indigenous Australians born in Australia (0.3% of 13 836 559, excluding people who inject drugs and men who have sex with men).^9^

Indigenous Australians also have a higher prevalence of comorbidities such as type 2 diabetes mellitus and alcohol-related liver disease^10^ which are associated with poorer prognosis and more rapid progression of chronic hepatitis B.^8^^,^^9^^,^^11^^,^^12^ The incidence of liver cancer is up to 10 times higher compared with non-indigenous Australians.^8^^,^^9^ In the light of this increased risk, indigenous Australians have been identified as a priority group for hepatitis B testing and immunization.^13^^–^^15^ Universal hepatitis B infant immunization is funded under the Australian national immunization programme, with a school-based adolescent catch-up programme funded to 2013 (by which time the cohort immunized in infancy will have reached adolescence). However, funding and access to hepatitis B vaccination for adults at higher risk, including indigenous Australians, is limited and inconsistent across the eight Australian states and territories (jurisdictions).^16^ Although data on hepatitis B vaccination coverage in indigenous adults are very limited, we believe that national coverage is likely to be low. This view is based on various evidence: from a sentinel study quantifying vaccination coverage from analysis of markers of hepatitis B infection;^17^ from the poor coverage achieved in funded influenza and pneumococcal vaccination programmes targeted on indigenous adults aged 18–49 years with relevant risk factors;^18^ and on the basis of the ongoing higher rate of incident infections in indigenous than non-indigenous adults.^19^

In this study, we aimed to review the epidemiology of newly acquired hepatitis B (the vaccine-preventable fraction of the burden of disease) in indigenous and non-indigenous people in Australia. We also estimated the potential impact of more systematic implementation of hepatitis B vaccination for all non-immune indigenous Australian adults.

## Methods

### Data sources

Data on all cases of hepatitis B notified from 1 January 2005 to 31 December 2012 were obtained from the Australian National Notifiable Diseases Surveillance System (NNDSS).^20^ The NNDSS compiles notification data from all eight Australian jurisdictions. Variables extracted for analysis included year of notification, age, sex, jurisdiction and indigenous status.

Data on the mid-year estimated national resident population for the same years (2005–2012) by jurisdiction; indigenous status and age were obtained from the Australian Bureau of Statistics.^1^

#### Definitions

Indigenous notifications included all individuals with hepatitis B notification whose indigenous status was recorded in the NNDSS as Aboriginal or Torres Strait Islander, or both; non-indigenous notifications included all other individuals with hepatitis B notification, including those whose indigenous status was unknown. The indigenous population was individuals who self-identified in the Australian census as being Aboriginal or Torres Strait Islander, or both (with the numbers adjusted for net undercount measured by a post-enumeration survey); non-indigenous was the remaining resident Australian population.

Since 2004 the national surveillance case definition of newly acquired (i.e. acute) hepatitis B is based on laboratory confirmation of infection by one of the following criteria: (i) detection of HBsAg in a patient shown to be negative within the last 24 months; or (ii) detection of HBsAg and of immunoglobulin (Ig) M to hepatitis B core antigen (anti-HBc IgM), in the absence of prior evidence of hepatitis B virus infection; or (iii) detection of hepatitis B virus by nucleic acid testing and of anti-HBc IgM, in the absence of prior evidence of hepatitis B virus infection.^21^ Confirmed cases of newly acquired hepatitis B are notifiable under public health legislation in each jurisdiction. Unspecified cases of hepatitis B (those with laboratory-definitive evidence but not meeting any of the criteria for a newly acquired case)^22^ are assumed to be predominantly episodes of chronic hepatitis B and are notifiable.

#### Ethical considerations

Ethics approval was not required for this study as de-identified, aggregate population-based data were used for routine public health purposes only.

### Data analysis

Analysis was restricted to newly acquired hepatitis B notification data. This was because the unspecified hepatitis B notification data do not differentiate between acute and chronic hepatitis B. Data were analysed by age group, sex, jurisdiction and indigenous status. While the completeness of recording indigenous status in notification data has improved, some variation exists between jurisdictions and over time. For this analysis, individuals whose indigenous status was unknown were classified as non-indigenous, according to established practice.^23^ To assess any impact of under-identification of indigenous status, a separate analysis was undertaken excluding data from jurisdictions that had completeness of recording indigenous status below 95%.

Australian Bureau of Statistics’ population data were used to calculate hepatitis B notification rates per 100 000 population and perform direct age standardization using the total Australian resident population as the standard.^1^ Rate ratios comparing notification rates for indigenous and other Australians were calculated with 95% confidence intervals (CI). Analyses were performed using Stata statistical software version 12.0 (Stata Corp., College Station, United States of America [USA]).

#### Vaccination impact estimates

The proportion of indigenous Australians aged 15 years or older who were non-immune, by age group, was estimated from Australian Bureau of Statistics’ population data^1^ and the authors’ (FB) expert opinion (Table 1). Expert opinion was based on a review of published HBsAg seroprevalence data^8^^,^^9^^,^^24^^–^^28^ and published^17^ and unpublished hepatitis B vaccination coverage estimates, and was informed by previous experience implementing immunization programmes targeting indigenous Australians. While hepatitis B vaccination coverage in indigenous Australian infants has been consistently high (in the vicinity of 95%) after universal immunization was introduced in the year 2000,^23^^,^^29^^,^^30^ coverage for adolescents was estimated to have been moderate and for adults was estimated to have been poor. We also accounted for the complexity involved in delivery of a hepatitis B immunization programme (including baseline serological testing and administration of three doses of vaccine).

**Table 1:** Values and probability distribution of model parameters for estimating the impact of a hepatitis B immunization programme for non-immune indigenous people aged ≥ 15 years in Australia

Age group, years	Estimated indigenous population, no.^a^	Estimated % susceptible (range)^b^	Estimated susceptible, no.	Estimated baseline no. of new infections per year^c^	Seroconversion rate from vaccination (range)^d^	Estimated risk of progression to chronic infection in newly acquired cases (range)^e^
15–19	72 782	30 (20–40)	21 835	23	0.95 (0.93–0.97)	0.10 (0.08–0.15)
20–24	61 166	40 (30–50)	24 466	44	0.90 (0.85–0.95)	0.08 (0.07–0.11)
25–29	50 390	40 (30–50)	20 156	29	0.90 (0.85–0.95)	0.08 (0.07–0.11)
30–34	40 681	40 (30–50)	16 272	21	0.90 (0.85–0.95)	0.07 (0.01–0.10)
35–39	41 300	40 (30–50)	16 520	33	0.90 (0.85–0.95)	0.07 (0.01–0.10)
40–44	40 507	40 (30–50)	16 203	18	0.75 (0.70–0.80)	0.07 (0.01–0.10)
45–49	34 189	40 (30–50)	13 676	14	0.75 (0.70–0.80)	0.07 (0.01–0.10)
50–54	28 812	40 (30–50)	11 525	3	0.65 (0.60–0.70)	0.07 (0.01–0.10)
55–59	21 562	40 (30–50)	8 625	3	0.65 (0.60–0.70)	0.07 (0.01–0.10)
60–64	15 190	40 (30–50)	6 076	1	0.65 (0.60–0.70)	0.07 (0.01–0.10)
65–69	9 680	40 (30–50)	3 872	1	0.40 (0.35–0.45)	0.07 (0.01–0.10)
70–74	5 972	40 (30–50)	2 389	3	0.40 (0.35–0.45)	0.07 (0.01–0.10)
≥ 75	7 030	40 (30–50)	2 812	0	0.40 (0.35–0.45)	0.07 (0.01–0.10)
**Total**	**429 261**	**NA**	**164 427**	**193**	**NA**	**NA**

NA: not applicable.

^a^ Estimated population from Australian Bureau of Statistics data.^1^

^b^ On the basis of author’s (FB) expert opinion informed by review of published data on seroprevalence of hepatitis B virus surface antigen^8^^,^^10^^,^^24^^–^^28^ and published data^18^ and unpublished estimates on hepatitis B vaccination coverage. While hepatitis B vaccination coverage in indigenous Australian infants has been consistently high (in the vicinity of 95%) after universal immunization was introduced in the year 2000,^23^^,^^29^^,^^30^ coverage for adolescents was estimated to have been moderate and for adults was estimated to have been poor.

^c^ Average annual number of notifications to the Australian national notifiable diseases surveillance system over the years 2005–2012, multiplied by 10.^31^^,^^32^

^d^ Point estimates were derived from the literature for areas with intermediate or low endemicity for adolescents^33^^–^^37^ and adults.^38^^–^^44^ The values of lower and upper limits were based on the author’s (FB) expert opinion.

^e^ Estimate from Edmunds et al. 1993.^45^

Notes: Risk of developing acute infection in susceptible individuals was calculated by dividing no. of newly acquired cases by age-specific population counts. Projected cumulative additional immunization coverage is presented in Table 2.

On this basis we estimated the potential impact of hepatitis B immunization for two vaccination coverage scenarios: (i) low coverage, in which 25% of the susceptible population of 164 427 were vaccinated by the end of the 10-year programme, and (ii) high coverage, in which 50% of the susceptible population was vaccinated. These figures correspond to 10% and 20% respectively of the total indigenous adult population. A range of possible coverage, determined by author’s (FB) expert opinion, was used for each of these scenarios, with cumulative coverage plotted for each year of the 10-year programme (Table 2; available at: http://www.who.int/bulletin/volumes/94/11/16-169524). We also used the model to estimate the vaccination coverage level at which hepatitis B incidence among indigenous Australians would be reduced to the current rate among non-indigenous Australians.

**Table 2:** Projected cumulative additional hepatitis B immunization coverage of susceptible indigenous people aged ≥ 15 years in Australia, over a 10-year immunization programme, for two vaccination coverage scenarios

Year	Projected % (range) of population vaccinated^a^	
	Low coverage scenario^b^	High coverage scenario^b^
1	5 (2–8)	20 (15–25)
2	8 (5–11)	24 (19–29)
3	11 (8–14)	28 (23–33)
4	13 (10–16)	32 (27–37)
5	15 (12–18)	35 (30–40)
6	18 (15–21)	38 (33–43)
7	21 (18–24)	41 (36–46)
8	25 (21–28)	44 (39–49)
9	25 (21–28)	47 (42–52)
10	25 (21–28)	50 (45–55)

^a^ For a completed course of three doses of vaccine; coverage was assumed to be the same across age groups (≥ 15 years); range applicable to Markov chain Monte Carlo model.

^b^ Low coverage scenario assumed 25% of the total susceptible indigenous adult population vaccinated after 10 years. High coverage scenario assumed 50% vaccinated.

Note: The estimated population size of susceptible indigenous Australians aged ≥ 15 years was 164 427.

We estimated the impact of a hepatitis B immunization programme for non-immune indigenous Australians aged ≥ 15 years in terms of the number of cases of acute and additional chronic hepatitis B infections prevented, and the number needed to vaccinate to prevent each case. To do this we developed a Markov chain Monte Carlo simulation using a random walk (i.e. a computerized run-through of 100 000 scenarios, each with variables randomly selected from the range of parameter values outlined in Table 1), using Excel 2010 software (Microsoft Corp., Redmond, USA). Our model was built on a Markov chain with one-year cycles and allowed for age-dependent transitions. This type of model structure conceptualizes health or disease status as a series of mutually exclusive and collectively exhaustive health states. In each cycle of the model, individuals either reside in one of the health states, or transition probabilistically to another at the end of a given cycle. Health benefits are accrued based on the state occupied during a given cycle. 

**Fig. 1:**
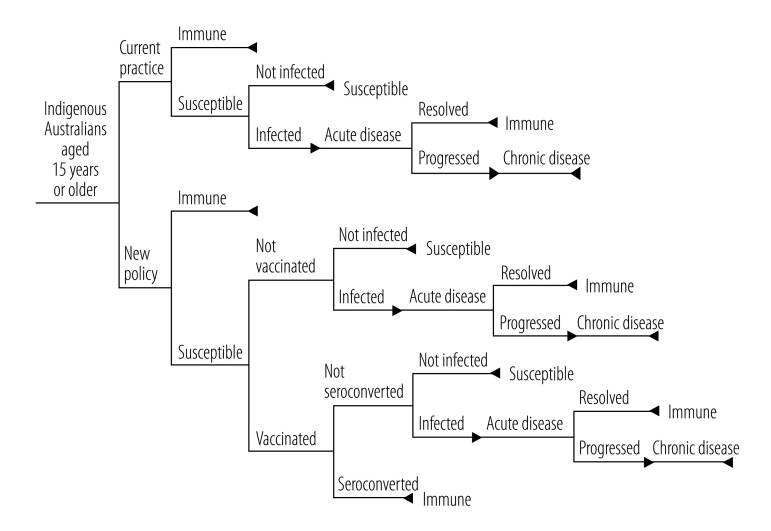
Decision tree used for estimating potential impact of hepatitis B immunization programme among indigenous people in Australia

A decision tree was used to compare the impact of a hepatitis B immunization programme targeting indigenous Australian adults, in terms of progression to acute or chronic infection, against the baseline of current practice (Fig. 1).

The variables incorporated into the model included: age group, estimated population, estimated proportion of people susceptible to hepatitis B, estimated baseline number of acute infections per year and vaccination seroconversion rate. Estimates of the risk of susceptible individuals developing acute infection and of progression to chronic infection were also incorporated into the model; these were derived from the literature from areas with intermediate or low endemicity for adolescents^33^^–^^37^ and adults.^38^^–^^40^^,^^44^

For the probability distribution of age group-specific parameters (Table 1), we assumed a normal distribution for the proportion of the population susceptible to hepatitis B infection, the seroconversion rate after vaccination and the projected cumulative additional vaccination coverage achieved in the hypothetical 10-year immunization programme. We used a triangular distribution for the risk of progression to chronic infection in newly acquired cases.

The key assumptions used in our model were as follows. Records of newly acquired cases in the NNDSS were considered as cases of acute hepatitis B virus infection. To adjust for underreporting and misclassification of acute hepatitis B infection, we used previously modelled data from Australia (similar to estimates from the USA)^31^^,^^46^ to estimate the true number of acute infections. We did this by applying a multiplier of 10 to the average annual number of NNDSS notifications of newly acquired hepatitis B for each age group over the period 2005–2012. Vaccine efficacy was considered all-or-nothing rather than leaky (partial protection). Vaccine efficacy for specific age groups was assumed to be equivalent to the seroconversion rate derived from overseas studies, as this is a well-established and robust surrogate of clinical protection.^32^ Infected individuals or seroconverted vaccinees were assumed to stay immune indefinitely. Finally, individuals younger than 15 years at the programme start, but who subsequently entered the model when they reached 15 years of age during the modelled programme, were assumed to be immune due to high levels of vaccination coverage in this cohort.

The potential effects on herd protection and mother-to-child transmission were not factored into the model. Any additional vaccination coverage achieved over the 10-year period through existing mechanisms (characterized by inconsistent funding and poor promotion) was assumed to be low and was not factored into the model.

## Results

### Notifications

There were 54 522 notifications of hepatitis B disease between 1 January 2005 and 31 December 2012, 52 569 (96%) of which were recorded as unspecified and 1953 (4%) as newly acquired. Of the newly acquired infections, 156 (8%) were recorded as being in indigenous persons. The overall notification rate over eight years in indigenous persons was 3.6 per 100 000 population (156/4 368 511) compared with 1.1 per 100 000 (1797/168 449 302) in the non-indigenous population. The age-standardized rate ratio for newly acquired hepatitis B for all ages from all jurisdictions over the study period was 4.0 (95% CI: 3.7–4.3) for indigenous Australians (Table 3). For the three jurisdictions with the highest (≥ 95%) completeness of recording indigenous status (Western Australia, South Australia and the Northern Territory), the age-standardized rate ratio was 4.7 (95% CI: 4.0–5.5). The notification rate ratio for indigenous Australians compared with other Australians over the study period were significantly higher in all age groups ≥ 15 years, ranging from 3.4 (95% CI: 2.4–4.6) in the 30–39 years age group to 7.3 (95% CI: 4.1–12.5) in the 15–19 years age group (Table 3).

**Table 3:** Notification rates for newly acquired hepatitis B virus infections (total 1953) and rate ratios, by age group and indigenous status, Australia, 2005–2012

Age group, by indigenous status^a^	Population, no.	No. of notifications	Notification rate per 100 000 population^b^	Rate ratio (95% CI)
**0–14 years**				
Indigenous	1 576 636	4	0.3	2.6 (0.7–7.5)
Non-indigenous	31 579 216	30	0.1	
**15–19 years**				
Indigenous	481 829	18	4.2	7.3 (4.1–12.5)
Non-indigenous	11 273 295	61	0.6	
**20–29 years**				
Indigenous	719 869	58	9.1	3.9 (2.9–5.1)
Non-indigenous	24 204 768	514	2.4	
**30–39 years**				
Indigenous	572 789	43	7.8	3.4 (2.4–4.6)
Non-indigenous	24 137 421	529	2.3	
** ≥ 40 years**				
Indigenous	1 017 388	33	3.5	4.3 (3.0–6.2)
Non-indigenous	77 254 602	663	0.8	
**All ages (age-standardized)**				
Indigenous	NA	NA	4.3	4.0 (3.7–4.3)^c^
Non-indigenous	NA	NA	1.1	

CI: confidence interval; NA: not applicable.

^a^ Indigenous population was individuals who self-identified in the Australian census as being Aboriginal or Torres Strait Islander or both (with the numbers adjusted for net undercount measured by a post-enumeration survey); non-indigenous was the remaining resident Australian population. Indigenous notifications included all individuals with hepatitis B notification whose indigenous status was recorded in the Australian national notifiable diseases surveillance system as Aboriginal or Torres Strait Islander or both; non-indigenous notifications included all other individuals with hepatitis B notification, including those whose indigenous status was unknown.

^b^ Age-specific average annual rate of notifications.

^c^ Rate ratio for all ages calculated using direct age standardization with all Australians as the standard.

**Fig. 2:**
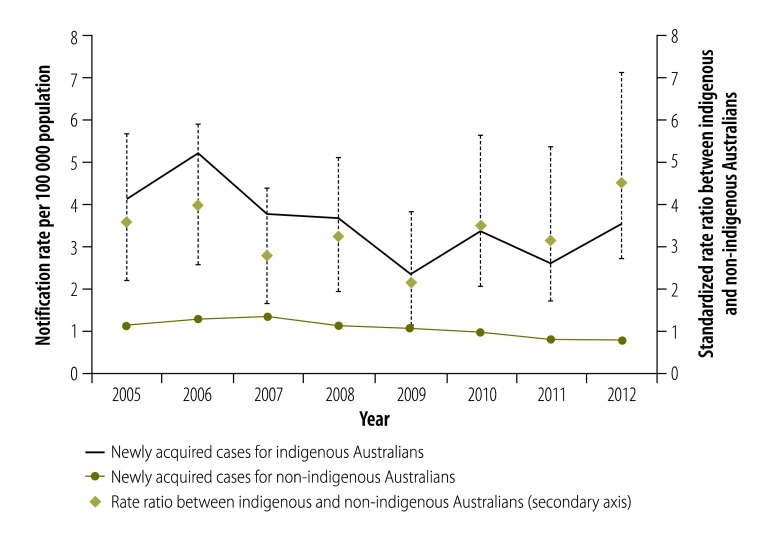
Trends in notification rates of newly acquired hepatitis B (left axis) and corresponding age-standardized rate ratios (right axis) by indigenous status, Australia, 2005–2012

Note: Rate ratios were calculated using direct age standardization with the total Australian resident population as the standard.

Notification rates for newly acquired hepatitis B between 2005 and 2012 showed no significant changes over time for indigenous Australians (Fig. 2). However, there was a significant downward trend in the annual notification rate for non-indigenous Australians, falling from 1.1 per 100 000 population in 2005 to 0.8 per 100 000 in 2012 (*P* < 0.001). The annual rate ratios for newly acquired hepatitis B showed no significant change over the period (Fig. 2).

### Vaccination impact estimates

We estimated the size of the hepatitis B non-immune indigenous Australian population aged ≥ 15 years to be approximately 164 000 (38% of the total indigenous population aged ≥ 15 years of 429 261; Table 1). 

With no additional adult vaccination coverage above that already occurring, modelling predicted an additional 1792 new acute hepatitis B cases in indigenous individuals aged ≥ 15 years over a 10-year period.

**Fig. 3:**
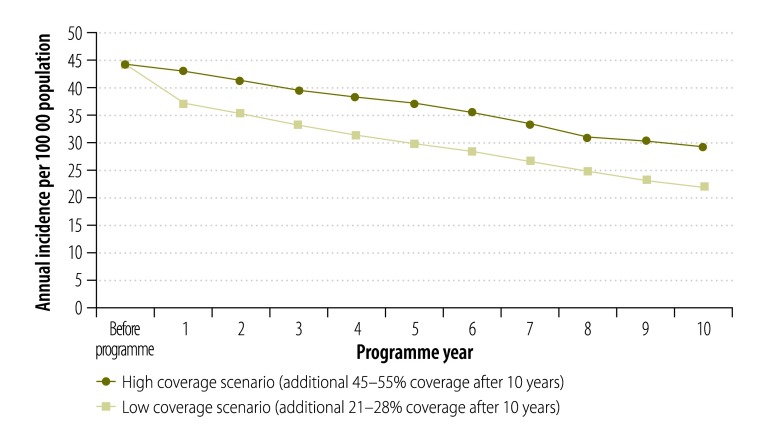
Projected annual trends in acute hepatitis B incidence over a 10-year immunization programme among indigenous people aged ≥ 15 years in Australia, by vaccination coverage scenario

Note: Vaccination coverage scenario in susceptible individuals was for completed course of three doses of vaccine and excluding impact of herd immunity. Low coverage scenario assumed 25% of the total susceptible indigenous adult population vaccinated after 10 years. High coverage scenario assumed 50% vaccinated.

Potential health gains for each scenario are summarized in Table 4; the projected annual trends in acute hepatitis B incidence over a 10-year immunization programme are shown in Fig. 3. In the first scenario, whereby 25% (range: 21–28%) of susceptible indigenous adults are vaccinated, the model predicted between 240 and 251 new cases of acute hepatitis B would be prevented across 10 years. The corresponding number of persons needed to vaccinate to prevent one case of acute hepatitis B under this scenario would be between 149 and 181 (Table 4). In the second scenario whereby 50% (range: 45–55%) of susceptible indigenous adults are vaccinated over a 10-year period, between 527 and 549 new cases of acute hepatitis B were predicted to be prevented, with an estimated number needed to vaccinate of between 138 and 163 (Table 4).

**Table 4:** Projected impact on number of acute and chronic cases of hepatitis B in a 10-year immunization programme for indigenous people aged ≥ 15 years in Australia, by vaccination coverage

Vaccination coverage	Acute hepatitis B^a^		Chronic hepatitis B^b^	
	No. of cases prevented (95% UI)	No. of people needed to vaccinate (UI)^c^	No. of cases prevented (95% UI)	No. of people needed to vaccinate (UI)^c^
Low coverage scenario^d^	246 (240–251)	164 (149–181)	23 (22–24)	1776 (1565–2009)
High coverage scenario^d^	538 (527–549)	150 (138–163)	50 (48–52)	1620 (1460–1793)

UI: uncertainty interval.

^a^ Newly acquired infection.

^b^ Persistent infection (hepatitis B surface antigen positive for ≥ 6 months post-infection).

^c^ Estimates of lower and upper limits for Markov chain Monte Carlo model. Number needed to vaccinate were calculated using bounds of 95% confidence interval (CI) of modelled number of cases prevented and bounds of 95% CIs of modelled number of vaccinees.

^d^ Projected coverage across a 10-year immunization programme. Low coverage scenario assumed 25% of the total susceptible indigenous adult population vaccinated after 10 years. High coverage scenario assumed 50% vaccinated.

Note: The estimated population size of susceptible indigenous Australians aged ≥ 15 years was 164 427.

For chronic hepatitis B the projected numbers of cases prevented were proportionately lower and the number needed to vaccinate were higher for both scenarios (Table 4).

## Discussion

Consistent with a previous study,^23^ we found that rates of notification for newly acquired hepatitis B were significantly higher for indigenous than non-indigenous Australians. Low rates of acute hepatitis B infection in both indigenous and non-indigenous Australians younger than 15 years^23^ reflect the success of the universal infant hepatitis B immunization programme, which began in Australia in 2000, building on targeted programmes during the preceding decade.

While current Australian guidelines recommend hepatitis B vaccination to be offered to all non-immune indigenous Australians, vaccination for indigenous adults is not funded under the national immunization programme and current uptake is thought to be poor. Our study shows that a hepatitis B immunization programme for indigenous Australians aged ≥ 15 years could have considerable impact in terms of cases of acute hepatitis B prevented, with a relatively low number of persons needed to vaccinate to prevent each case. Prevention of acute hepatitis B infection would also have an impact on the ultimate number of cases of chronic infection. Without such a programme, it will almost certainly take several decades for the disparity in rates of acute hepatitis B infection between indigenous and non-indigenous Australian adults to reduce, as those vaccinated as infants gradually age into adulthood.

As of 2008, 177 countries had introduced hepatitis B vaccine into their national infant immunization programmes.^47^ This is estimated to have prevented more than 80% of the 1 400 000 hepatitis-B-related deaths that would otherwise have occurred worldwide since WHO’s initial recommendation in 1997.^48^ Our findings support WHO’s recommendation that catch-up campaigns for hepatitis B vaccination be considered for adolescents or adults in high-prevalence settings once infant immunization is established.^47^ Modelling similar to that conducted in our study could be used to estimate the impact of catch-up programmes in such settings or in high-risk populations in intermediate- or low-prevalence settings. The benefits of catch-up hepatitis B strategies for higher-risk populations in countries with intermediate or low endemicity are also reflected in the WHO guidelines for the prevention, care and treatment of persons with chronic hepatitis B infection;^49^ so too is a particular focus on offering screening, vaccination and treatment for hepatitis B to indigenous peoples. For example, a catch-up hepatitis B immunization programme for adolescents aged 15–19 years was estimated to be cost saving in China^50^ and was subsequently implemented with good effect.^51^ Another campaign targeting young adults aged 21–39 years in China has also been estimated to be cost saving.^51^

Our study had several limitations. We did not use a dynamic model, primarily due to the lack of data on contact mixing patterns applicable to the indigenous population in Australia. The static Markov chain Monte Carlo method we used is not able to capture herd protection effects, which could be substantial considering the well-documented household overcrowding and heightened disease transmission that occurs within indigenous communities.^52^ Also, we did not evaluate any potential incremental benefits on prevention of mother-to-child transmission. This route of transmission is uncommon in Australia, including in indigenous populations, due to the high quality of antenatal care and neonatal immunization. Nor did we estimate the protection afforded to additional individuals who may receive an incomplete course of vaccinations. Grouping notifications from people with unknown indigenous status with non-indigenous status likely underestimates the true disparity between indigenous and non-indigenous populations.^53^ Lower access to health care may also contribute to underestimation of notification rates for indigenous Australians. However, this may be counteracted by recommendations in national guidelines to test all indigenous Australians for hepatitis B virus infection. We did not factor in any impact of vaccination coverage achieved through existing mechanisms over the 10-year period as this was assumed to be low. On balance, these limitations are likely to result in our estimates of potential impact being conservative.

Our study findings highlight the health disparity in hepatitis B infection between indigenous and non-indigenous Australians. This is connected to the overall disadvantage faced by indigenous Australians, which is due to a complex combination of inter-related socioeconomic, cultural and historical determinants.^4^ It is also likely that hepatitis B was highly prevalent in the indigenous population before infant immunization programmes began. Multiple initiatives at many levels by the Australian government have been put in place to address the broader issues of indigenous disadvantage. The findings of our study suggest that this disparity in hepatitis B could be readily and rapidly eliminated through a modest increase in vaccination coverage among indigenous Australian adults, for example through a funded vaccination catch-up programme.
